# *PCSK9* Variants in Familial Hypercholesterolemia: A Comprehensive Synopsis

**DOI:** 10.3389/fgene.2020.01020

**Published:** 2020-09-23

**Authors:** Qianyun Guo, Xunxun Feng, Yujie Zhou

**Affiliations:** Beijing Key Laboratory of Precision Medicine of Coronary Atherosclerotic Disease, Department of Cardiology, Beijing Anzhen Hospital, Clinical Center for Coronary Heart Disease, Beijing Institute of Heart Lung and Blood Vessel Disease, Capital Medical University, Beijing, China

**Keywords:** gene, genetics, proprotein convertase subtilisin/kexin type 9, familial hypercholesterolemia, variant

## Abstract

Autosomal dominant familial hypercholesterolemia (FH) affects approximately 1/250, individuals and potentially leads to elevated blood cholesterol and a significantly increased risk of atherosclerosis. Along with improvements in detection and the increased early diagnosis and treatment, the serious burden of FH on families and society has become increasingly apparent. Since FH is strongly associated with proprotein convertase subtilisin/kexin type 9 (*PCSK9*), increasing numbers of studies have focused on finding effective diagnostic and therapeutic methods based on *PCSK9*. At present, as *PCSK9* is one of the main pathogenic FH genes, its contribution to FH deserves more explorative research.

## Introduction

A hereditary propensity for elevated serum levels of low-density lipoprotein cholesterol (LDL-C) that leading to cardiovascular disease (CVD) is typical FH and affects approximately 1 in 250 individuals. Currently recognized FH-inducing variants that lead to disease occur mostly in the apolipoprotein B (*APOB*), *PCSK9*, and LDL receptor (*LDLR*) genes. While most cases are caused by *LDLR* variants, they may also be caused by autosomal dominant variants of *PCSK9*, although less frequently (Defesche et al., 2017; Viigimaa et al., 2018; Sarraju and Knowles, 2019). PCSK9 encodes the proprotein convertase subtilisin/kexin type 9 protein which binds LDL and regulates the numbers of LDLR. As the third gene implicated in FH, *PCSK9* has been found to reduce the uptake of LDL-C in the liver by increasing the endosomal and lysosomal degradation of *LDLR* (Maxwell and Breslow, 2005; Stoekenbroek and Kastelein, 2018). Moreover, experimental studies indicate that *PCSK9* might independently accelerate atherosclerosis by enhancing inflammation, endothelial dysfunction, and hypertension (Urban et al., 2013). *PCSK9* variants implicated in autosomal dominant hypercholesterolemia (ADH) were first identified in 2003. At the time, they were believed to induce abnormal cholesterol metabolism through undefined mediators. Since then, *PCSK9* variants have been extensively investigated. Among these, loss-of-function (LOF) variants are associated with reduced LDL-C levels and coronary artery disease (CAD) risk, while gain-of-function (GOF) variants diminish *LDLR* levels, thereby inducing hypercholesterolemia (Abifadel et al., 2009). To prevent morbidity and mortality, it is crucial to diagnose FH early. Predominantly, laboratory tests or medical traits including family history are used in diagnosis, however, genetic screening of pathological variants might make definite diagnoses more accessible (Hovingh et al., 2013). To explore *PCSK9* variants in FH and their biological roles, we provide a detailed discussion and summary of novel *PCSK9* variants. We also discuss the important interplay between *PCSK9* and other molecules. Finally, this review sums up novel treatment strategies aimed at *PCSK9* in FH to pave the way for future investigative studies.

## History of *PCSK9* Variant Discoveries

**FIGURE 1:**
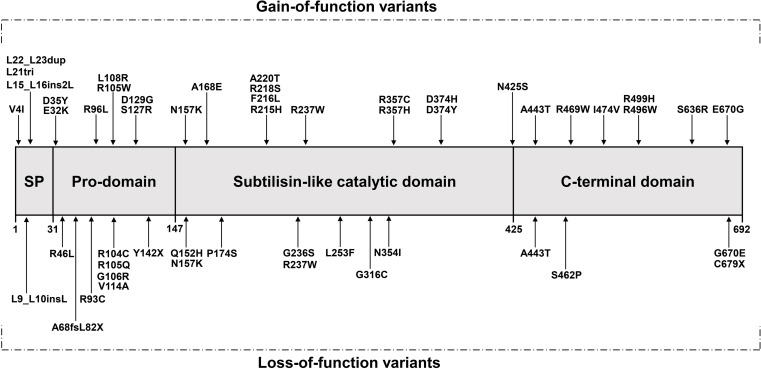
The protein’s domain structure of *PCSK9* and location of variants (adopted and redrawn from Maxwell and Breslow, 2005; Soutar and Naoumova, 2007) SP, Signal peptide.

Seventeen years ago, through the sequencing of 12 exons of the *PCSK9* gene, Abifadel et al. (2003) opened the door to researching the role of the *PCSK9* gene in FH which suggested a novel mechanism of dyslipidemia. They identified a T to A substitution in exon 2 at nucleotide 625 resulting in a non-synonymous change at codon 127 of arginine for the conserved serine p.(S127R) and the substitution of T to C, resulting in an amino acid substitution of phenylalanine to leucine p.(F216L) among different families. In the following 5 years, many studies have been carried out to discover new variants in *PCSK9* (Figure 1). In addition, different populations have different types and frequencies of *PCSK9* variants, so we also provide a summary table of *PCSK9* variants with their population frequencies and LDL-C levels from previous studies (Tables 1, 2).

**TABLE 1:** Summary of GOF variants among different populations.

Researchers	*PCSK9* variants	Populations	Sample size	Variant frequency, %	LDL-C ranges, mmol/L
Leren (2004)	D374Y	Norwegian, FH	51	5.9	7.0–10.6
Allard et al. (2005)	R357H	French, FH	130	0.8	4.3–6.2
	R469W	French, FH	130	0.8	6.0–9.2
Humphries et al. (2006)	D374Y	British, FH	409	1.7	1.82–6.77
Taylor et al. (2007)	D374Y	British, FH	400	2.2	>4.9
Bourbon et al. (2008)	D374H	Portuguese, FH and relatives	602	0.5	4.9–9.4
Noguchi et al. (2010)	E32K	Japanese, FH	55	6.4	5.8–8.8
Abifadel et al. (2012)	D35Y	French, ADH	75	2.7	6.0
Mabuchi et al. (2014)	E32K	Japanese, FH	1,055	5.9	8.0–16.0
Ohta et al. (2016)	V4I	Japanese, FH	269	6.3	4.5–7.8
	E32K	Japanese, FH	269	6.3	4.5–7.8
	R496W	Japanese, FH	269	0.4	4.5–7.8
Xiang et al. (2017)	R96L	Chinese, FH	219	0.5	4.5–12.2
	R105W	Chinese, FH	219	0.5	4.5–12.2
Kaya et al. (2017)	D374Y	Turkish, FH	80	5.0	2.2–6.5
	R496W	Turkish, FH	80	8.7	2.0–9.8
Eroǧlu et al. (2018)	D374Y	Turkish, dyslipidemia	200	7.0	3.7–8.7
	R496W	Turkish, dyslipidemia	200	6.5	3.7–8.7
Luirink et al. (2019)	A220T	Netherlander, FH	1,903	0.1	7.7–9.0

*Normal range of LDL-C: Low-risk groups < 3mmol/L; High-risk groups < 1.8 mmol/L; FH groups < 1.4 mmol/L. (According to the 2019 ESC/EAS guidelines for the management of dyslipidaemias). PCSK9, proprotein convertase subtilisin/kexin type 9; LDL-C, low-density lipoprotein cholesterol; FH, familial hypercholesterolemia; ADH, autosomal dominant hypercholesterolemia.*

**TABLE 2:** Summary of LOF variants among different populations.

Researchers	*PCSK9* variants	Population	Sample size	Variant frequency, %	LDL-C ranges, mmol/L
Cohen et al. (2006)	Y142X	ARIC study, general	3,363	0.8	1.7–3.7
	C679X	ARIC study, general	3,363	1.8	1.4–3.8
	R46L	ARIC study, general	9,524	3.2	2.2–3.9
Hooper et al. (2007)	C679X	African, general	653	3.7	1.3–1.9
Scartezini et al. (2007)	R46L	British, general	2,444	1.0*	1.8–4.0
Guella et al. (2010)	R46L	Italian, MI patients	1,880	1.0*	2.1–3.9
Chernogubova et al. (2012)	R46L	Swedish, general	5,722	1.9*	2.5–4.9
Saavedra et al. (2014)	R46L	Canadian, FH	582	3.0	5.8–7.7
Langsted et al. (2016)	R46L	CGPS study	103,083	1.3*	2.2–3.4
Mostaza et al. (2018)	R46L	Spanish, adults	1,188	2.9*	2.6–4.3
	R46L	Spanish, children and adolescents	1,933	3.2*	2.2–2.6

Normal range of LDL-C: Low-risk groups < 3 mmol/L; High-risk groups < 1.8 mmol/L; FH groups < 1.4 mmol/L. (According to the 2019 ESC/EAS guidelines for the management of dyslipidaemias). PCSK9, proprotein convertase subtilisin/kexin type 9; LDL-C, low-density lipoprotein cholesterol; ARIC, Atherosclerosis Risk in Communities; MI, myocardial infarction; CGPS, Copenhagen General Population. *Represents the allele frequency which is different from the PCSK9 variant proportion of the population in the different references.

### Variant Sites Identified Within the First 6 Years

#### Variants Related to Elevated Levels of LDL-C

In 2004, Timms et al. (2004) recognized a single G→T nucleotide variant present on the K1173 haplotype variant resulting in the non-synonymous p.(D374Y). Leren identified an asparagine to lysine substitution at position 157, p.(N157K) (Leren, 2004). Benjannet et al. (2004) subsequently identified two further *PCSK9* variants occurring naturally in the gene pool, namely p.(R218S) and p.(R237W). In addition, Shioji et al. (2004) demonstrated the association between total cholesterol (TC) and LDL-C levels with exon 9/I474V or intron 1/C(-161)T polymorphisms.

In 2005, the amount of plasma LDL-C and the extent of atherosclerosis in coronary arteries were shown by Chen et al. (2005) to depend on a single nucleotide polymorphism resulting in p.(E670G). Subsequently, Allard et al. (2005) found four heterozygous missense variations resulting in changes in *PCSK9*, namely p.(R218S), p.(R357H), p.(R469W), and p.(A443T) in the coding region and intronic junctions of the *PCSK9* gene after analyzing 130 patients with ADH.

From 2006 to 2008, Pisciotta et al. (2006) sequenced multiple genes including *PCSK9* in two patients with heterozygous *LDLR* genes who were diagnosed with homozygous FH (HoFH). They identified one patient with the p.(R496W) variant from her mother and another one with the p.(N425S) variant likely from her deceased father. Patients with mutated *LDLR* were speculated to suffer worse pathological symptoms if they had an uncommon missense *PCSK9* variant. One year later, Bourbon et al. (2008) identified a p.(D374H) variant of *PCSK9* in 184 patients and 418 relatives in Portugal, and found that the number of confirmed FH patients increased through cascade screening. They recommended that if patients received appropriate treatment to restrain progress of premature CAD after early identification of FH, their life expectancy and quality of life could be improved. FH patients from New Zealand were observed to have two novel missense variants, namely p.(D129G) and p.(A168E) and together with two established variants from South Africa, namely p.(S127R) and p.(R237W), these gave a *PCSK9* variant total of four discovered by Homer et al. (2008) They found that the inhibition of *LDLR* mediated by *PCSK9* occurred independently of *PCSK9* release or autocatalytic destruction and speculated that *PCSK9* might play a role in cells. In addition, five novel *PCSK9* variants were found by Cameron et al. (2008), including p.(R215H), p.(G236S), p.(N354I), p.(A245T), and p.(R272Q), with p.(R215H) resulting in GOF and hypercholesterolemia. Since their effect on the internalization of LDL-C was similar to that of the wild-type (WT) *PCSK9*, it was demonstrated that p.(R272Q) and p.(A245T) were non-pathological aberrations which maintained normal *PCSK9* performance, though both variants were identified in a hypercholesterolemic group. After studying the promoter variant of the *PCSK9* gene, Blesa et al. (2008) identified a c.-332C > A variant in the region of the *PCSK9* gene promoter that increased transcription of PCSK9. In fact, the variant could result in a 2.5-fold increase in transcription compared to WT, thereby leading to ADH. At the end of the year, Abifadel et al. (2008) found familial combined hyperlipidemia present in two families, with variants of two leucines [designated p.(L15_L16ins2L) and p.(L21tri)] in family members who also had elevated LDL-C, thus suggesting an association.

#### Variants Associated With Decreased Levels of LDL-C

Cohen et al. (2005) investigated 128 people whose plasma LDL-C was low and identified two nonsense variants including p.(C679X) and p.(Y142X) through determining the coding region sequence of *PCSK9*. These had been recognized as LOF variants since 2005 as they had opposite effects compared to the GOF *PCSK9* variants. Subsequently, in 2006, while examining 38 people with hypocholesterolemia and 25 heterozygotes with hypercholesterolemia, all from different families, Berge et al. (2006) screened for *PCSK9* variants. They identified four variants including p.(R46L), p.(G106R), p.(R237W), and p.(N157K) among the two groups. In the same year, Kotowski et al. (2006) employed oligonucleotide hybridization on a chip and deoxyribonucleic acid (DNA) sequencing to identify p.(L253F), p.(A443T), and p.(R46L) missense mutants which were significantly correlated with diminished amounts of LDL-C. Moreover, after sequencing for variations in 403 Caucasians, Yue et al. (2006) identified a c.43_44insCTG mutant that mediated LDL-C level reductions in normal people. In 2007, Fasano et al. (2007) identified a novel variant in exon 1 (c.202delG) of a single nucleotide deletion from one heterozygous patient, that resulted in messenger ribonucleic acid (mRNA) generating peptide frameshift and truncation at p.(A68fsL82X). In the next year, Miyake et al. (2008) found 33 *PCSK9* gene sequence variants, with one at p.(R93C) which had a 0.051 R93C allele prevalence in low vs. high LDL-C groups, and was associated with low LDL-C levels. Meanwhile, the LOF variants p.(G236S) and p.(N354I) were described by Cameron et al. (2008). The p.(G236S) variant prevented *PCSK9* release from the endoplasmic reticulum (ER) while p.(N354I) resulted in failure of *PCSK9* to undergo autocatalytic cleavage.

### Identification of Novel *PCSK9* Variants Associated With FH in the Last 10 Years

In the past 10 years, with the emergence of related studies on *PCSK9* inhibitors and other treatment of patients with statin-intolerant hyperlipidemia, including patients with FH, less attention has been paid to the analysis of *PCSK9* variants than previously (Figure 1).

With respect to LOF variants of *PCSK9*, Cameron et al. (2009) identified the novel variant p.(S462P) in exon 9 of the *PCSK9* gene and suggested that this variant, like the p.(G236S) and p.(N354I) variants, prevented normal C-terminal domain folding precluding release of the protein from the ER. Another LOF variant, the double-mutant p.(R104C)/p.(V114A), which improved the clearance rate of LDL-C (Cariou et al., 2009). Mayne et al. (2011) identified a p.(Q152H) substitution in a French-Canadian family which resulted in a 48% reduction in LDL-C concentration compared with non-carriers. Slimani et al. (2012) investigated *PCSK9* and *LDLR* variants in Tunisian FH families, identifying a new missense variation p.(P174S) that appeared to cause decreased levels of LDL-C with respect to the LDLR genotype in six family members. In Pakistan, FH family members who carried the p.(R105Q) variant had lower levels of total cholesterol suggesting that this variant might cause LOF (Ahmed et al., 2013). Through targeted next-generation sequencing (NGS), the variants p.(R93C) and p.(G670E) were identified by Lee et al. (2017) in nine patients with very low levels of LDL-C. In addition, one carrier of the heterozygous missense variant p.(G316C) that was associated with hypocholesterolemia and steatosis was found (Di Filippo et al., 2017).

Regarding *PCSK9* GOF variants, two novel variants were found in 75 patients with ADH and normal *APOB* and *LDLR* genes by Abifadel et al. (2012) namely p.(D35Y) and p.(L108R) substitutions. The variants were absent from individuals with normal cholesterol levels and were associated with the presence of ADH in families. The authors also assessed the quantitative and qualitative effects of these *PCSK9* variants on lipoprotein granules and their effect on the activity of cholesterol ester transfer protein (CETP). This was the first report of the impact of *PCSK9* variants on high density lipoprotein (HDL)-mediated cholesterol release from cells. Ohta et al. (2016) found that the p.(V4I) variant of *PCSK9* in 269 clinically diagnosed FH heterozygotes was related to a remarkably increased CAD prevalence accompanied by increasing levels of LDL-C, although the levels of serum lipids and the CAD prevalence between p.(V4I) carriers and non-carriers without *LDLR* variants remained similar. Di Taranto et al. (2017) demonstrated two GOF variants, p.(S636R) and p.(R357C), considered to cause FH. In their study, the p.(S636R) and p.(R357C) variants showed a lower binding capacity for WT *PCSK9* than *LDLR*. They also found a further two mutants with uncharacterized effects on FH disease progression. Elbitar et al. (2018) sequenced exons from 13 French FH families and discovered a *PCSK9* variant at p.(R96C) in a patient with a severe phenotype from a family with the p.(A3396T) *APOB* variant. They demonstrated that this was the first reported compound heterozygote having both *APOB* and *PCSK9* variants. In addition, Sánchez-Hernández et al. (2019) found a novel *PCSK9* variant, p.(R499H) of in two unrelated FH patients from Spain and Italy. This variant resulted in decreased expression of *LDLR* from *in vitro* functional assays. There were two related studies on *PCSK9* in FH children: DECOPIN and GoTCHA. In the DECOPIN project, Ibarretxe et al. (2018) identified 49 different variants including two in *PCSK9* parents and children with FH. In the child-to-parent study, p.(R496W) and p.(L22_L23dup) were found to be pathogenic in families. Luirink et al. (2019) analyzed a total of 1903 FH children with molecular assays in the GoTCHA study. They also conducted candidate gene sequencing in HoFH children whose LDL-C levels were above the lowest level measured in pediatric patients with HoFH. In their study, a GOF variant of *PCSK9* p.(A220T) was found in two related patients.

In studies of the *PCSK9* variants in Asian populations, Noguchi et al. (2010) identified the p.(E32K) variant in Japanese people. In their study, the frequency of the p.(E32K) variant in clinical FH was significantly higher than that in a control group (6.42 vs. 1.71%). Although this GOF variant might have milder effects than the p.(D374Y) and *LDLR* variants, it could worsen lipid conditions in true homozygous or double heterozygous probands with *LDLR* variants. In China, Xiang et al. (2017) identified two *PCSK9* variants in FH patients including p.(R96L) and p.(R105W) from the central southern region of China. As GOF variants, these two variants might cause increased *LDLR* degradation, resulting in a decrease of LDL-C clearance, eventually giving rise to hypercholesterolemia. In their study, they also found another nine novel variants which included seven *LDLR* variants and two *APOB* variants.

## Molecular and Biological Mechanisms to Explore the Influence of *PCSK9*

**FIGURE 2:**
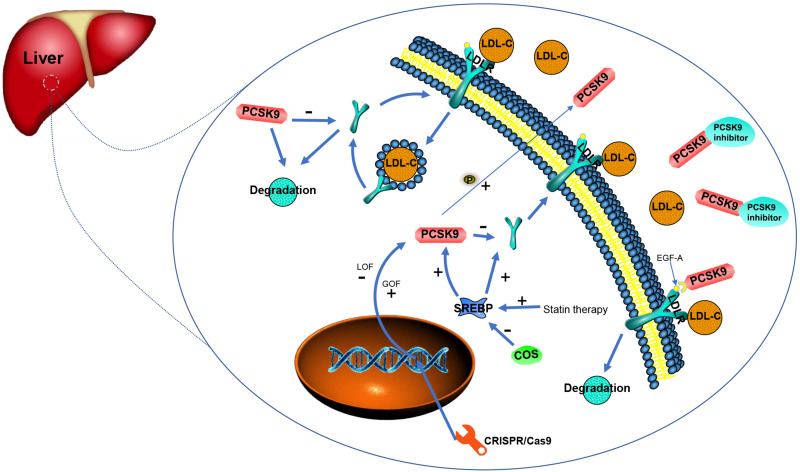
Function and regulation related to *PCSK9* and existing/potential therapies for *PCSK9* inhibition (adopted and redrawn from Hovingh et al., 2013; German and Shapiro, 2020).

*PCSK9* belongs to the proprotein convertase family of serine proteases. Biological and gene analyses have shown that *PCSK9* is a vital regulator of *LDLR* proteins which in turn, regulate plasma LDL-C. Through indirectly causing the degradation of *LDLR*, *PCSK9* normally downregulates *LDLR* with LOF *PCSK9* variants resulting in low levels of LDL-C in the plasma (Soutar and Naoumova, 2007; Ding and Kullo, 2008; Schmidt et al., 2008; Figure 2).

### Interactions Between *PCSK9* and Its Related Proteins

#### Influential Elements in *PCSK9* Transcription

Conversion of *PCSK9* is regulated by the sterol regulatory element-binding protein (SREBP) 2, a membrane transcription factor. In addition, transcription to enable *PCSK9* production is also regulated by SREBP 2 (Jeong et al., 2008; Wiciński et al., 2017). GOF variants in *PCSK9* have been found proven to lead to FH through reducing *LDLR* protein expression in the liver and decreasing the clearance of circulating cholesterol. Statin therapy is able to inhibit cholesterol biosynthesis followed by the activation of SREBP to increase the expression of *LDLR*. However, statins also induce the synthesis of *PCSK9*, leading to the degradation of *LDLR* (Rashid et al., 2005; Tall, 2006). Transcription of *PCSK9*, either basal or sterol-regulated, depends on the recognition of a motif in the promoter region by histone nuclear factor P (HINFP). Variants in this region prevent sterol-induced blockade and diminish promoter activity at basal levels in addition to abating promoter activation by SREBP 2 (Li and Liu, 2012). Transcription factors for SREBP depend on binding to the SREBP cleavage-activating protein (SCAP) in the ER membrane. Investigations of SCAP inhibition in a monkey model revealed that when SCAP small interfering RNA (siRNA) encapsulated in lipid nanoparticles is added, both LDL-C levels and *PCSK9* are significantly reduced (Murphy et al., 2017). Chitin oligosaccharides (COS) have antioxidant and anti-inflammatory activities and have been recently found to suppress *PCSK9* gene expression thereby decreasing the number of LDLRs on the cell surface. Thus, down-regulation of SREBP2 by COS decreases the expression of *PCSK9* (Yang et al., 2018).

#### Connection Between *PCSK9* and *LDLR*

*PCSK9* binds to the epidermal growth factor precursor homology domain A (EGF-A) on extracellular *LDLR* domains which regulates hepatic *LDLR*s and leads to their degradation. Asp-374 on the surface of the subtilisin-like catalytic domain of *PCSK9* binds the *LDLR* EGF-A domain. Substitution of the Asp with Tyr (the p.(D374Y) GOF variant enhances *PCSK9* affinity for *LDLR*. This combination of *PCSK9* and *LDLR* may cause a conformational change in *LDLR* that prevents the recycling of *LDLR* from the plasma membrane, instead of leading the complex containing *PCSK9* and *LDLR* to degradation (Kwon et al., 2008; Cariou and Dijk, 2020; Martin et al., 2020). Effects on the folding of *PCSK9* potentially caused by LOF variants such as p.(Q152H), could trigger increased clearance of LDL-C in the circulation, due to the reduction of *LDLR* degradation mediated by *PCSK9* (Garvie et al., 2016). In tandem, researchers have found that the 314–355 *LDLR* EGF-A domain and the 153–421 *PCSK9* catalytic domain are involved in the interaction between *PCSK9* and *LDLR* that leads to decreased *LDLR* levels and LDL-C accumulation (Alghamdi et al., 2015). In a strategy using synthetic EGF-A analogs, it has been found that the peptide with the greatest potency enhanced *PCSK9* binding affinity compared with WT EGF-A (Schroeder et al., 2014). In addition, a study of the C-terminal domain of *PCSK9* created seven *de novo* mutants of *PCSK9* and investigated their affinity toward a calcium-independent mutant of the EGF-A domain. This showed that the p.(G517R), p.(V644R), and p.(V610R) mutants have descending abilities to prevent LDL-C growth in HepG2 cells (Geschwindner et al., 2015). Moreover, ligand-binding (LR) repeats of *LDLR* have been identified for *PCSK9*-mediated *LDLR* degradation and the p.(D203N) variant in the LR5 of full-length LDLR was found to significantly reduce *PCSK9* binding (Deng et al., 2019). Interestingly, in studies of post-translational modifications, *PCSK9* treatment can also result in ubiquitination of *LDLR*. Importantly, if the *LDLR* protein carried variants in its C-terminal ubiquitination sites, it was able to resist *PCSK9*-mediated degradation (Chen et al., 2011). Recently, it has been observed that phosphorylation may enhance the secretion of *PCSK9* from hepatocytes, thus maximizing *LDLR* degradation through both extracellular and intracellular pathways (Ben Djoudi Ouadda et al., 2019).

### Possible Effects of *PCSK9* on Cellular Function

The lectin-like ox-LDL receptor-1 (LOX-1) has been shown to play a critical role in inflammatory diseases, including atherosclerosis, where LOX-1 and *PCSK9* positively influence each other’s expression and it appears that mitochondria-derived reactive oxygen species (mtROS) may to be important initiators of *PCSK9* and LOX-1 expression (Ding et al., 2015). Interestingly, LOX-1 and *PCSK9* might be upregulated secondary to induction by ox-LDL in a concentration-dependent manner and ox-LDL-induced human umbilical vein endothelial cell death could be inhibited by *PCSK9* siRNA (Wu et al., 2012). Subsequently, in a study of vascular smooth muscle cell mitochondrial DNA (mtDNA) and (SMC)-derived *PCSK9* damage, it was found that in the presence of mtROS, there was a positive relationship between mtDNA damage and SMC-derived *PCSK9*. This interaction leads to cell damage, characterized by apoptosis (Ding et al., 2016). Meanwhile, endothelial cell apoptosis may be repressed through mitogen-activated protein kinase signaling in atherosclerosis by shRNA-*PCSK9* targeting of *PCSK9* (Li et al., 2017). Investigation of *PCSK*-induced autophagy mechanisms showed that *PCSK9* might be up-regulated in ischemic hearts, thus determining infarct size, cardiac function, and autophagy development through the activation of the ROS-related axis (Ding et al., 2018).

## From Diagnosis Methods to Therapy Strategies of FH Based on *PCSK9*

### Cascade Screening

Cascade screening is a vital procedure for identifying people at risk for inherited diseases. For some autosomal dominant diseases, such as FH, relatives can be identified for significant health-affecting interventions, thus significantly increasing life expectancy. Cascade screening is an evidence-based intervention that has been found to reduce cardiovascular morbidity and mortality in the FH population (Knowles et al., 2017). A series of population-based screening and research initiatives, represented by the Dutch Lipid Clinic Network in the Netherlands (DLCNC), have made tremendous progress in raising awareness and treatment of FH (O’Brien et al., 2014). When cascade screening was first introduced in the Netherlands, 2039 relatives of 237 FH cases were found to have FH, 39% of whom had already been receiving treatment. An Australian study also found that among 100 relatives of genetically diagnosed FH patients, 51.4% had pathogenic variants identified by cascade screening. In other recent cases, they have demonstrated other CAD risk factors and have already started using statins without prior diagnosis of FH (Bell et al., 2015; Henderson et al., 2016). In Asia, cascade screening is proving to be an efficient method for the diagnosis of FH in Vietnamese family members. After screening 107 relatives in five FH patients, 56 cases were diagnosed with FH, including three HoFH cases (Truong et al., 2018). Cascade screening is also important in children and adolescents. After analyzing 292 children with FH from 205 parents, researchers found that 20 percent of the parents and 49 percent of the maternal grandparents had an early history of CVD. Similarly, in a Slovenian study, nearly every child diagnosed with FH had a parent who was at high risk for FH. Thus, CVD is still the main cause of FH, and despite the absence of evidence on the long-term safety of drug therapy in childhood, genetic natural history studies confirm the benefits of lifelong low LDL-C levels, so early initiation of cascade screening will facilitate early intervention in the next generation (Galema-Boers et al., 2015; Wiegman et al., 2015; Groselj et al., 2018).

Early detection and treatment of FH in individuals and families could help reduce the morbidity and mortality associated with FH. Indeed, the setting up of an FH database and registration system would be a critical measure to enhance the long-term outcomes of FH patients. Currently, in most countries, diagnosis and treatment models are not adequate (Singh and Bittner, 2015; Chen et al., 2019). However, the cost of DNA sequencing in patients has fallen considerably in the last few years and if the rate of progress continues, the current lack of detection and screening might change (Louter et al., 2017). Below, we list the essential and potential ways of FH detection and screening.

### Innovative Ways of Detecting and Screening in FH

#### From Sanger Sequencing to NGS

Before NGS was widely applied, the genetic diagnosis of FH mainly relied on Sanger sequencing to identify variants in the *APOB* and *PCSK9* genes. After overcoming many of the scalability barriers faced by clinical laboratories using traditional Sanger methods when performing large-scale DNA sequencing according to the guideline and list of genes reported as incidental or secondary findings of the American College of Medical Genetics (ACMG), NGS has gradually been proved to be a reliable and practical molecular screening method for FH pathogenic genes and become one of the main techniques for *PCSK9* detection, while Sanger sequencing is mainly used as a verification method to assist the accuracy of NGS detection (Rehm et al., 2013; Rimbert et al., 2016; Kalia et al., 2017; Pek et al., 2018). Moreover, the guidelines for diagnostic NGS of the EuroGentest and European Society of Human Genetics have emphasized that, although NGS testing was still being explored and developed, NGS technology offered potential overall benefits for the diagnosis of patients’ diseases (Matthijs et al., 2016). As NGS are increasingly used for routine FH diagnosis, FH-related variants may be identified exponentially, so detection of disease-related variants in FH patients is critical for early intervention to reduce the risk of CVD. In a study of a British cohort, compared with multiplex polymerase chain reaction and oligonucleotide arrays, the NGS method has shown great analytical performance with approximately 89–100% concordance to other methods (Reiman et al., 2016; Iacocca et al., 2018). What is more, in the first reported NGS test for variants in clinically suspected FH patients in Singapore, the percentage of detected variants was similar to that of western countries, and although no *PCSK9* variants were found, it indicated that NGS technology covering all exons of *LDLR* might be a better strategy (Pek et al., 2018).

In the 2020 technical standard of ACMG, it is recommended that the laboratory must consider the effectiveness of NGS analysis and augment NGS testing with ancillary assays (Bean et al., 2020). In a study of NGS as a potential method for diagnosing FH, NGS-based testing has been shown to involve lower cost and less labor than traditional sequencing genetic testing. This may provide a way to increase the genetic diagnosis of the current low proportion of FH (Norsworthy et al., 2014). With the continuous development of NGS technology, new NGS-based detection is also gradually being applied to FH screening. In a single target NGS panel study, this new NGS is found to be an effective variant detection method, which can better help to understand the phenotype of FH, and is expected to become a personalized diagnosis method for dyslipidemia (Marmontel et al., 2018). In addition, in the first study of capture-based NGS, this NGS method covering the entire *LDLR* genome region has improved the efficiency of structural variation detection. This method is expected to comprehensively detect the pathogenic variants of *LDLR*, *APOB*, and *PCSK9* in FH patients (Hsiung et al., 2018).

#### iPLEX Test

To relieve the shortage of intensive procedures which have complicated genetic diagnoses, Wright et al. (2008) developed the Multiplex MassARRAY Spectrometry (iPLEX) and identified 56 variants in a number of genes, including one variant in *PCKS9*, in DNA samples from 92 FH patients. From this study, it is clear that, while the FH iPLEX test is aimed at screening for FH variants in large-scale targeted populations, it is also suitable for population screening. The Agena iPLEX designed by Chiou and Charng (2017). has been found to have higher specificity and sensitivity to FH gene screening, compared with the traditional diagnostic Sanger sequencing procedure, in detecting DNA from 120 FH patients with defined molecular causes.

#### HRM Method

In contrast to existing detection methods for genetic abnormalities in FH patients, a high-resolution melting (HRM) analysis known as the polymerase chain reaction (PCR) with modifiable melting conditions, has been proven to be more efficient compared to DNA sequencing. Firstly, it is more cost-effective and timesaving. Secondly, as a sensitive and robust technique, it is capable of detecting new sequence changes that would make sense in cascade screening of HF subjects (Liyanage et al., 2008; Whittall et al., 2010; Pećin et al., 2013). In a Turkish FH cohort using HRM analysis on isolated DNA, it was demonstrated that two *PCSK9* GOF variants [p.(D374Y) and p.(R496W)] in FH have a higher frequency and different courses of disease compared to other populations around the world (Kaya et al., 2017).

#### Chips Technology

Given the large number of patients with suspected FH, molecular genetic analysis of the entire genome is time-consuming. A new diagnostic tool in the form of a genotyping DNA microarray chip called FH chip based on arrayed primer extension (APEX), has been proposed to accelerate variant screening in Czech FH patients. In this study, researchers found that the validation phase of this FH chip had 100% sensitivity and 99.1% specificity. They suggested that the FH chip could be implemented for genotyping with features of rapidity, reproducibility, specificity, and economy (Dušková et al., 2011). The Belfast Genetics Laboratory aims to keep abreast of evolving technology by simple and economic genetic testing. An FH biochip array protocol is followed by using samples analyzed for FH variants prior to incorporation into cascade screening. Study results estimate that NGS is five times more expensive than the cost of testing and reporting one sample through the FH Biochip technique due to the latter’s high sensitivity and rapid detection ability (Martin et al., 2016).

### Current Therapies for *PCSK9-*Associated FH

#### *PCSK9* Inhibitors

Appropriate medical treatment is essential to effectively manage FH, including reducing cardiovascular risk and improving the prognosis of affected patients. The current drug treatments for FH mainly include statins, bile acid-binding resins, and cholesterol absorption inhibitors. Considering that the risk of cardiovascular events in FH patients is significantly increased, timely reduction of LDL-C is essential to reduce the risk of CVD. However, traditional drugs may have difficulty in achieving the goal of decreased blood lipid in FH patients, thus, *PCSK9* inhibitors with strong lipid-lowering effects have gradually become a new class of drugs for the treatment of FH (Fala, 2016; Papademetriou et al., 2018). In a meta-study, it was found that when evolucomab is given as a 420 mg monthly dose, LDL-C could be reduced by 54.71%, indicating that evolocumab might be a potential drug for FH patients (Eslami et al., 2017). It has also been found that alirocumab treatment is well-tolerated in heterozygous FH (HeFH) patients and could significantly reduce LDL-C by 12 and 24 weeks while evolocumab could effectively reduce the LDL-C levels in HoFH or severe HeFH patients over a median of 4.1 years (Ginsberg et al., 2019; Santos et al., 2020). In summary, *PCSK9* inhibitors can significantly reduce LDL-C, even in FH patients who have not yet achieved their LDL-C goals. Therefore, to ensure that FH patients can receive the *PCSK9* inhibitor drug therapies, it is necessary to increase the diagnosis rate and conduct family screening in certain populations (Ogura, 2018).

#### Inclisiran

In recent years, with the advent of siRNA, researchers have designed a new generation of drugs to combat *PCSK9* named Inclisiran. It can reduce the concentration of *PCSK9* in the body by interfering *PSCK9* gene expression in hepatocytes with a double-stranded short sequence of RNA, thereby reducing the degradation of *LDLR* and enhancing the ability of hepatocytes to eliminate LDL-C to reduce its levels (Dyrbuś et al., 2020). In 2017, Ray et al. (2017) found that Inclisiran could significantly reduce LDL-C after the first subcutaneous injection (>50%) and maintained this level for up to 1 year after the first injection. Compared with the placebo group, the Inclisiran group had no serious adverse reactions. In the subsequent phase III clinical study of Inclisiran, compared with placebo, LDL-C in Inclisiran group was significantly reduced, with the efficacy able to last for more than 18 months (Raal et al., 2020; Ray et al., 2020). The introduction of *PCSK9* inhibitors is a milestone in the treatment of FH, and Inclisiran provides a new lipid-lowering technology. The clinical trials of the Inclisiran series of drugs are expected to produce a revolutionary new lipid-lowering drug (German and Shapiro, 2020).

### Development of CRISPR/Cas9 Therapy in *PCSK9*

In 2003, the characterization of *PCSK9*’s LDL-C regulatory functions resulted in a landmark paradigm shift in therapies for hypercholesterolemia (Seidah et al., 2019). There is reason to believe that it might soon be possible to achieve effective cholesterol management by permanently and selectively modifying the genome and inactivating the function of target genes with a single injection (Fazio and Tavori, 2014). Among the nine ways to realize *PCSK9* inhibition, clustered regularly interspaced short palindromic repeats (CRISPR) technology might have great potential (Mullard, 2017). Though the CRISPR/Cas9 technology offers flexibility for treating hyperlipidemia and is capable of changing the genome to permanently decrease cholesterol levels, more research is needed before their application in human subjects (Banerjee et al., 2016; Chadwick and Musunuru, 2017).

#### The Attempt and Exploration

Ding et al. (2014) use CRISPR/CRISPR-associated (Cas) technology to obtain a *PCSK9*-targeting CRISPR guide RNA and Cas 9 expression in murine livers with an adenovirus to effectively disrupt murine *PCSK9* genes, resulting in reduced levels of cholesterol and *PCSK9*, but elevated amounts of liver *LDLR*. This momentous finding in genome editing might have potential for the therapy and prevention of CVD in humans. Subsequently, severely diminished levels of blood *PCSK9* are seen in mice with humanized livers with almost 50% of highly specific human hepatocyte variants, thus demonstrating the safety and effectiveness of CRISPR-Cas9 in reducing human *PCSK9* levels (Wang et al., 2016). In addition, CRISPR/Cas9-mediated genome editing decreases *PCSK9* levels in both human and murine hypercholesterolemic models, which could be a valuable tool in the search for novel therapeutic approaches against hypercholesterolemia (Carreras et al., 2019).

#### The Development and Outlook

It is to get into mammalian cells without a vehicle. Additionally, gene editing using CRISPR/Cas9 technology, which is both sizeable and based mainly on DNA, mRNA, or protein, also poses a significant challenge. Although viral vectors have higher delivery efficiency and adeno-associated viral vectors have recognized efficiency in atherosclerosis research, their biosecurity issues hinder their wide application. Based on the nanocarrier-delivered CRISPR/Cas9 system, Zhang et al. (2019) use a triple targeting strategy to produce a LOF variant in the *PCSK9* gene and this strategy might be a potential target therapy for CVD without side effects (Jarrett et al., 2018). In addition, precise knock-in of specific nucleotide changes have proven to be inefficient in non-proliferating cells *in vivo*. Chadwick et al. (2017) therefore, used *PCSK9* base editing, which has the ability to generate alterations in genes without the need for breaks in double-stranded DNA. This demonstrates the ability to precisely introduce therapeutically relevant nucleotide variants into the genome in somatic tissues in adult mammals.

## Recently Discovered Potential Genetic Targets Related to FH

### Presumptive Loci Related to FH

In order to identify ADH disease loci besides *PCSK9*, *LDLR* and *APOB*, on the basis of a genome-wide scan and linkage analysis, Marques-Pinheiro et al. (2010) report a large lineage from France and hypothesized the involvement of a fourth gene, named *HCHOLA4*, at 16q22.1. In their study, it is shown that other ADH genes do exist, while they also identify nine affected families with no linkage to the *HCHOLA4* locus nor the three known genes. Apart from *PCSK9*, *LDLR*, and *APOB*, it has been found that there is a significant relationship between ADH and rs965814 G allele mapping to 8q24.22 through genome-wide analysis on 15 family members (Cenarro et al., 2011).

### The Controversial Relationship Between *STAP1* and FH

Exome sequencing was performed by Fouchier et al. (2014) along with parametric linkage analysis in a family with FH4 (ADH with unmutated genes for *PCSK9*, *APOB*, and *LDLR*) to identify a fourth ADH relevant locus, leading to the mapping of the ADH locus at 4p13. In addition, ADH associates with four signal-transducing adaptor family member 1 (*STAP1*) variants, including p.(E97D), p.(L69S), p.(I71T), and p.(D207N). Out of all the *STAP1* missense variants analyzed by bioinformatics analysis and available structural information in one man and his two siblings, p.(P176S) which might play a role of affecting cholesterol homeostasis has been associated with FH as the likely most damaging variant (Blanco-Vaca et al., 2018). However, after *STAP1* was analyzed in hypercholesterolemic patients from Germany, whose variants are negative in canonical FH genes, it was concluded that the positive predictive value of *STAP1* analysis would be comparably small and in order to characterize *STAP1* contributions to FH pathogenesis, its molecular interactions should be explored through *in vitro* functional studies (Danyel et al., 2019). Moreover, despite noting that FH patients carrying *STAP1* have lower LDL-C levels than non-carriers, Lamiquiz-Moneo et al. (2020) found no phenotypic penetrance of their genome when exploring potential associations between phenotype and *STAP1* variants. What is more, studies of mouse models and carriers of *STAP1* variants, indicate that *STAP1* might not be the FH or LDL-C modulating gene and should not be considered for FH genetic screening (Loaiza et al., 2020).

## Perspective

The unique mechanism of action of *PCSK9* and the identification of its genetic variants have brought a new therapeutic target to FH. Among the numerous variant sites, both GOF and LOF might become new breakthrough points of treatment. Since the use of *PCSK9* inhibitors has emerged outside conventional FH treatment strategies, their gradually increasing applications in clinical practice have not only brought good news to FH patients but also new hope for patients with hyperlipidemia that are at high risk of CAD. It is reasonable to believe that, with continuous research progress on *PCSK9* in FH, more therapeutic methods and diagnostic methods with superior accuracy, safety, and economy will be applied in FH patients. Interactions of *PCSK9* with various cellular components, based on its unique features and influence on the body, are also worthy of further research and discussion. Last, but not least, as a disease related to genetic variant, there are undoubtedly more undiscovered genetic loci related to FH that are worth exploring in addition to the existing FH genes.

## Author Contributions

QG and XF wrote this review and YZ revised it. All authors read and approved the final manuscript.

## Conflict of Interest

The authors declare that the research was conducted in the absence of any commercial or financial relationships that could be construed as a potential conflict of interest.

## Abbreviations

ADH: autosomal dominant hypercholesterolemia
APEX: arrayed primer extension
APOB: apolipoprotein B
CAD: coronary artery disease
Cas: CRISPR/CRISPR-associated
CETP: cholesterol ester transfer protein
COS: chitosan oligosaccharides
CRISPR: clustered regularly interspaced short palindromic repeats
CVD: cardiovascular disease
EGF-A: epidermal growth factor precursor homology domain A
ER: endoplasmic reticulum
FH: familial hypercholesterolemia
GOF: gain-of-function
HDL: high density lipoprotein
HeFH: heterozygous FH
HINFP: histone nuclear factor P
HoFH: homozygous FH
HRM: high-resolution melting
LDL-C: low-density lipoprotein cholesterol
LDLR: LDL receptors
LOF: loss-of-function
LOX-1: lectin-like ox-LDL receptor-1
LR: ligand-binding
iPLEX: Multiplex MassARRAY Spectrometry
mRNA: messenger ribonucleic acid
mtDNA: mitochondrial DNA
mtROS: mitochondria-derived reactive oxygen species
NGS: next-generation sequencing
PCR: polymerase chain reaction
PCSK9: proprotein convertase subtilisin/kexin type 9
SCAP: SREBP cleavage-activating protein
SERBP: sterol regulatory element–binding protein
siRNA: small interfering RNA
STAP1: signal-transducing adaptor family member 1
TC: total cholesterol
WT: wild-type.
